# Expression Atlas update—an integrated database of gene and protein expression in humans, animals and plants

**DOI:** 10.1093/nar/gkv1045

**Published:** 2015-10-19

**Authors:** Robert Petryszak, Maria Keays, Y. Amy Tang, Nuno A. Fonseca, Elisabet Barrera, Tony Burdett, Anja Füllgrabe, Alfonso Muñoz-Pomer Fuentes, Simon Jupp, Satu Koskinen, Oliver Mannion, Laura Huerta, Karine Megy, Catherine Snow, Eleanor Williams, Mitra Barzine, Emma Hastings, Hendrik Weisser, James Wright, Pankaj Jaiswal, Wolfgang Huber, Jyoti Choudhary, Helen E. Parkinson, Alvis Brazma

**Affiliations:** 1 European Molecular Biology Laboratory, European Bioinformatics Institute, EMBL-EBI, Hinxton, UK; 2 Welcome Trust Sanger Institute, Hinxton, UK; 3 Oregon State University, Corvallis, USA

## Abstract

Expression Atlas (http://www.ebi.ac.uk/gxa) provides information about gene and protein expression in animal and plant samples of different cell types, organism parts, developmental stages, diseases and other conditions. It consists of selected microarray and RNA-sequencing studies from ArrayExpress, which have been manually curated, annotated with ontology terms, checked for high quality and processed using standardised analysis methods. Since the last update, Atlas has grown seven-fold (1572 studies as of August 2015), and incorporates baseline expression profiles of tissues from Human Protein Atlas, GTEx and FANTOM5, and of cancer cell lines from ENCODE, CCLE and Genentech projects. Plant studies constitute a quarter of Atlas data. For genes of interest, the user can view baseline expression in tissues, and differential expression for biologically meaningful pairwise comparisons—estimated using consistent methodology across all of Atlas. Our first proteomics study in human tissues is now displayed alongside transcriptomics data in the same tissues. Novel analyses and visualisations include: ‘enrichment’ in each differential comparison of GO terms, Reactome, Plant Reactome pathways and InterPro domains; hierarchical clustering (by baseline expression) of most variable genes and experimental conditions; and, for a given gene-condition, distribution of baseline expression across biological replicates.

## INTRODUCTION

Expression Atlas (2) is a further development of its predecessor, Gene Expression Atlas (1) launched by the European Bioinformatics Institute (EMBL-EBI) in 2008, and continues its original remit as a value-added database for querying gene expression across tissues, cell types and cell lines under various biological conditions. These include developmental stages, physiological states, phenotypes and diseases, and covers nearly 30 organisms including metazoans and plants. Expression Atlas is developed with a view to accommodating data from multi-omics experiments; the first proteomics data set has been included in 2015.

High-quality microarray and RNA-sequencing (RNA-seq) data in Expression Atlas continue to come from ArrayExpress (3), which also includes data imported from NCBI's Gene Expression Omnibus (GEO) (4). Expression is reported for both coding and non-coding transcripts. The sample attributes and experimental variables are carefully curated, systematized and mapped to the Experimental Factor Ontology (EFO (5)) for efficient search via ontology-driven query expansion, and to facilitate data integration with other resources.

Expression Atlas consists of two components—(i) a large baseline expression component (http://www.ebi.ac.uk/gxa/baseline/experiments), reporting transcript abundance estimates for each gene in healthy or untreated tissues, cell types or cellular components from carefully selected large RNA-seq experiments and (ii) information about the changes in transcript abundance between two different conditions, such as normal and disease.

Since the last update, we have included in the baseline Atlas a number of important projects such as Human Protein Atlas (8) and The Genotype-Tissue Expression (GTEx) project (7). New funding sources and user feedback have accelerated the expansion of Atlas into disparate data domains, for example plants and cancer. For the first time, Atlas contains 389 experiments studying plants in 11 species (http://www.ebi.ac.uk/gxa/plant/experiments), e.g. rice, wheat, maize and *Arabidopsis*, including 7 baseline studies reporting expression in tissues, strains and cultivars. 97 differential and 3 baseline experiments in Atlas study cancer.

Atlas’ ability to display expression across all tissues and all baseline studies next to each other in a single, intuitive interface makes it easy for the user to spot corroborating patterns of expression across multiple ‘omics studies. All differential expression data are now also available for further analysis as R objects.

Annotations reported by expression studies may become defunct due to addition of new transcribed loci or dropping invalid entries in updated genomic references. To address this, Atlas release cycle is synchronised with that of Ensembl (21), Ensembl Genomes (22) (including Ensembl Plants) and the Gramene (23) databases, guaranteeing the latest gene annotations, microarray probe-set mappings and genomic references. For each new genome assembly, all RNA-seq data in Atlas in the corresponding organism are re-processed to match the most current version of the reference genome. Recent examples of using Expression Atlas data for novel research include, for example, references (26–28).

## RESULTS

### Data

At the time of writing, Expression Atlas contains highly curated data from 1572 studies (69239 assays), incorporating RNA-seq based baseline expression (http://www.ebi.ac.uk/gxa/baseline/experiments, 36376 assays) in tissues from Human Protein Atlas (http://www.ebi.ac.uk/gxa/experiments/E-MTAB-2836), GTEx (http://www.ebi.ac.uk/gxa/experiments/E-MTAB-2919), FANTOM5 (10, http://www.ebi.ac.uk/gxa/experiments/E-MTAB-3358), in cancer cell lines from ENCODE (9, http://www.ebi.ac.uk/gxa/experiments/E-GEOD-26284), Cancer Cell Line Encyclopaedia (CCLE, 11, http://www.ebi.ac.uk/gxa/experiments/E-MTAB-2770) and Genentech (12, http://www.ebi.ac.uk/gxa/experiments/E-MTAB-2706) projects, as well as differential expression for manually curated comparisons (4287 as of August 2015). Table 1 shows the top 15 organisms in Atlas with the highest number of studies. Examples of plant data in Atlas include several studies of rice salt stress, for example time-course experiments studying *Oryza sativa japonica* cv. Nipponbare (salt-sensitive) variety: http://www.ebi.ac.uk/gxa/experiments/E-MTAB-1625 (RNA-seq) and http://www.ebi.ac.uk/gxa/experiments/E-MTAB-1624 (microarray), allowing for comparison of expression obtained from the same physical samples using different technologies. This line was chosen because the reference rice genome was also sequenced from it. Users can also view the baseline expression profile of genes from a gene family or a given pathway from Plant Reactome Wikipathways. For example, Figure 4 shows rice auxin efflux (*PIN*) and auxin influx (*AUX*) gene family members participating in Auxin (*IAA*) transport pathway in a plant cell.

**Figure 1.**
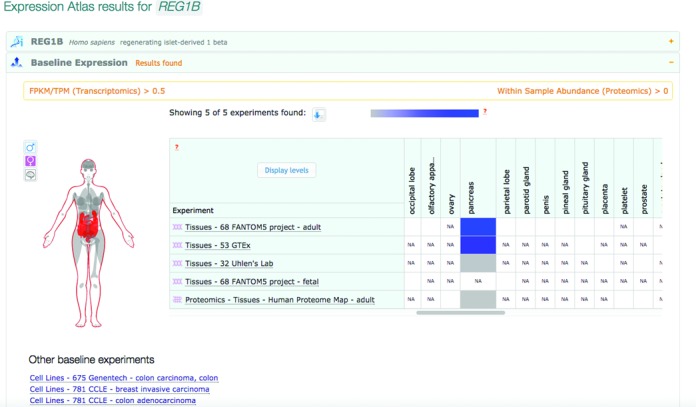
Baseline expression for human REG1B gene, corroborating high level of expression in pancreas across studies: FANTOM5, GTEx, Human Protein Atlas and a Proteomics study: a draft map of the human proteome, in http://www.ebi.ac.uk/gxa/genes/ENSG00000172023. The unit used for reporting expression in RNA-seq studies is FPKM, and in the proteomics study—the ‘within sample abundance’. ‘NA’ means that the tissue was not assayed in a given study.

**Figure 2.**
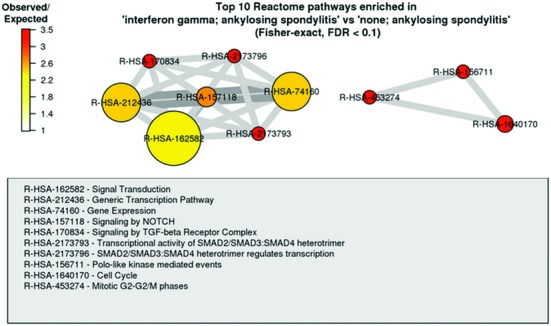
Top 10 Reactome pathways enriched in the set of genes differentially expressed in the comparison of ‘interferon gamma; ankylosing spondylitis’ versus ‘none; ankylosing spondylitis’ in http://www.ebi.ac.uk/gxa/experiments/E-GEOD-11886. Two distinct groups of pathways with are visible, with thicker edges between the pathways corresponding to the greater number of shared genes, and the pathways with the highest enrichment effect size (odds-ratio) shown in red.

**Figure 3.**
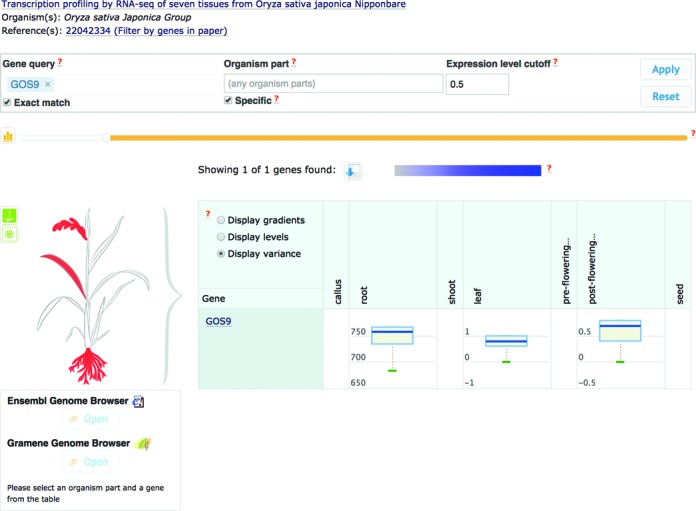
Variance of baseline expression across biological replicates in each tissue for rice gene GOS9: http://www.ebi.ac.uk/gxa/experiments/E-MTAB-2037?geneQuery = GOS9 (Please check the ‘Display variance’ radio button to see the box plots).

**Figure 4.**
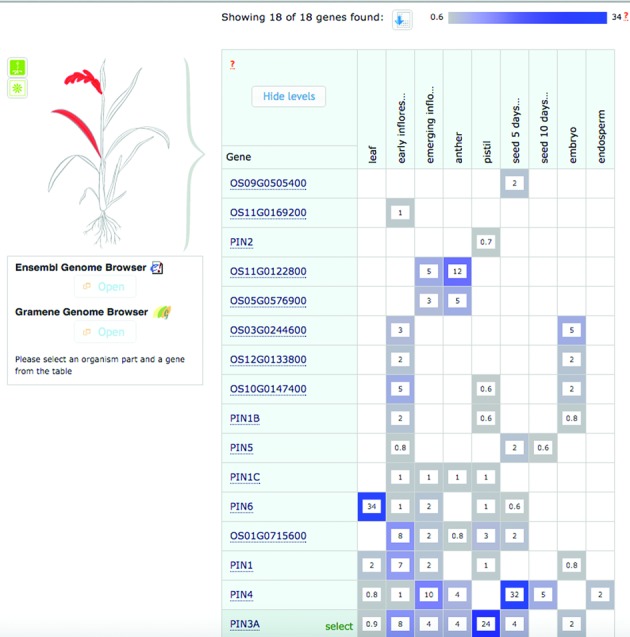
Baseline expression profile of gene family members participating in Auxin (*IAA*) transport pathway in a plant cell (http://wikipathways.org/index.php/Pathway:WP2940); http://www.ebi.ac.uk/gxa/experiments/E-MTAB-2039?geneQuery=OS01G0643300%09OS01G0715600%09OS01G0802700%09OS01G0856500%09OS01G0919800%09OS02G0743400%09OS03G0244600%09OS05G0447200%09OS05G0576900%09OS06G0232300%09OS06G0660200%09OS08G0529000%09OS09G0505400%09OS10G0147400%09OS11G0122800%09OS11G0137000%09OS11G0169200%09OS12G0133800.

**Table 1.** Top 15 organisms in Atlas—by the number of studies

Organism	Number of differential studies	Number of baseline studies
Mus musculus	496	10
Homo sapiens	477	8
Arabidopsis thaliana	341	1
Drosophila melanogaster	63	0
Rattus norvegicus	57	2
Saccharomyces cerevisiae	19	0
Oryza sativa Japonica Group	16	2
Caenorhabditis elegans	11	2
Gallus gallus	9	2
Zea mays	9	0
Sus scrofa	7	0
Danio rerio	6	0
Vitis vinifera	5	0
Bos taurus	4	2
Oryza sativa Indica Group	4	0
Others	11	8

Expression Atlas is intended as a multi-omics, and in particular as a functional genomics and proteomics, resource. Since both the transcript and peptide molecules undergo their own independent modifications as well as degradation in a spatial temporal manner, providing both kinds of data provides an opportunity for researchers to asses spatial temporal and condition based correlation of transcript amount versus the amount of its translated product estimated from proteomics experiments. While the quantitation and statistical analysis of transcript expression methods are relatively mature and well established, the equivalent methods for protein detection, quantification and statistical analysis are still active areas of research. Consequently, in the first instance, we have included our first protein expression data (http://www.ebi.ac.uk/gxa/experiments/E-PROT-1) as additional information to the transcriptomics data in the baseline component of Expression Atlas only, shown side by side for the corresponding tissues. This proteomics study consists of re-analysed mass spectrometry raw data from the draft map of the human proteome (25), downloaded from the PRIDE (6) repository (PXD000561), and comprising 85 experimental samples from 30 human adult and fetal tissues.

### Analysis

Since the last update, we have adopted Tophat2 (17) and HTSeq (15) for genome reference alignment and gene expression quantification respectively, for all RNA-seq experiments in Atlas. We have currently suspended reporting baseline expression for splice variants for several reasons—first, uncertainty about the reliability of the methods currently available (29), second, careful research has shown that for most genes in most conditions there is one dominant isoform expressed (20), and finally because of the high computational requirements.

Expression Atlas continues to analyse and report statistically significant differential gene expression in manually curated differential pairwise comparisons between two sets of biological replicates—the ‘reference’ (e.g. ‘healthy’ or ‘wild type’) set and a ‘test’ set (e.g. ‘diseased’ or ‘mutant’). The differential analysis is now performed using DESeq2 (18) with independent filtering (19). Since the last update we have also included parameterization of additional factors and blocking effects where possible, thus eliminating technical sources of variation and boosting statistical power in studies with heterogeneous sample sets. Consequently, we were able to load into Atlas 50 new studies, including clinical ones containing detailed patient histories.

Users now have more tools at their disposal to assess the accuracy of expression data reported by Atlas: (i) the number of biological replicates is now reported for a given baseline condition, or on either side of a differential comparison; (ii) for a given baseline condition, quantile normalisation is used to make distributions of expressions in each biological replicate the same—prior to averaging gene expression levels across biological replicates; (iii) for a given gene-condition, users can view a box plot of variability of baseline expression across biological replicates, providing them with an impression of how representative the reported median expression level is.

Expression Atlas remains committed to the stringent quality control of raw experimental data and design, reported in the previous update. Since then we have automated quality control of RNA-seq data, that involves exclusion of corrupted FASTQ files and those with an insufficient number of reads after filtering for poor quality and contamination. As in the case of microarray outlier array removal, removing poor quality data files may lead to the exclusion of affected differential comparisons, baseline conditions or even whole experiments from Atlas.

The proteomics data analysis methods are described in the Human Proteome Label Free Analysis section of the supporting material.

### New user interface features

Expression Atlas search interface allows for querying one or more genes or proteins from a selected species. The user can also add search filters for sample attributes and experimental factors, taking full advantage of ontology-driven query expansion. For example, searching for disease lymphoma will return expression data from samples of not only lymphoma itself, but also from its subtypes and closely related diseases, e.g. Hodgkin's lymphoma or acute myelogenic leukemia. Using the same interface, both baseline and differential components of Expression Atlas are queried by default. The Atlas interface displays search results from all tissues and all baseline studies making it possible to find patterns of expression across a wide variety of studies (Figure 1). We are working to extend this functionality to other types of experimental conditions for which Atlas has wealth of baseline expression data, e.g. cell lines, as well as to comparative views of expression, highlighting common tissue expression patterns for orthologues—across all available baseline data sets. The interface also showcases more detailed anatomical images, in which tissues with reported expression are highlighted. This now includes a separate brain diagram for human and mouse, as well as ‘whole plant’ and ‘flower parts’ diagrams for plant experiments.

Various novel analyses and visualisations have been implemented in Atlas. For example, the overlap between the set of differentially expressed genes in each Atlas comparison, and Reactome and Plant Reactome pathways, GO terms and InterPro domains is now assessed using Fisher's test with multiple testing correction (16). The resulting pathways, terms or domains that are ‘enriched’ in a given comparison are shown in network-style visualisations, including the effect size (Figure 2). For each baseline study, a visualisation of hierarchical clustering between the 100 most variable genes and experimental conditions is also shown. Finally, for a given gene-condition, the user can view a box plot of variability of baseline expression across biological replicates (Figure 3).

Expression data from Atlas are now viewable as tracks on Ensembl, Ensembl Genomes and Gramene genome browsers. Baseline expression data from Atlas are also automatically included in Ensembl, Ensembl Genomes, Gramene Ensembl Plants, Reactome and Plant Reactome, via javascript-based widgets. The widgets are easily accessible (https://github.com/gxa/atlas/blob/master/web/src/main/javascript/heatmap/README.md) and can be integrated in any third-party site, provided the bioentity identifiers match those of the Atlas.

## FUTURE DIRECTIONS

### New RNA-seq studies

We plan to include in Atlas the latest data from ENCODE, GTEx version 5, Blueprint (http://dcc.blueprint-epigenome.eu/), NIH Epigenomic Roadmap (13) and HipSci (http://www.hipsci.org/).

### Protein expression

A number of new proteomics studies will be loaded into Atlas in the near future.

### On-the-fly gene set ‘enrichment’

Users will be able to perform on-the-fly overlap analysis between their provided set of genes and differentially expressed gene sets in each comparison in Atlas, resulting in a (sorted by effect size) list of comparisons in which the user provided gene set is ‘enriched’.

### Gene co-expression

For a given gene within a single study, we will enable the user to find other genes of similar expression profile across experimental conditions or differential comparisons.

### Expression of orthologues

We plan to make available baseline expression of orthologues in tissues.

### Quantification of expression of exons and splice-variants

We plan to provide exon quantifications for all RNA-seq experiments. We will also re-visit the topic of splice-variant expression quantification by benchmarking several splice-variant expression quantification methods (namely, Kallisto and RSEM (14)), with the plan to bring splice variant quantification back into Atlas once the computational and accuracy issues are resolved satisfactorily.

### Analysis and visualisation of single-cell RNA-seq data

We plan to extend our analysis pipelines and visualisation methods to adequately annotate, quality control and visualise gene expression data from single-cell RNA-seq studies.

### Handling of blocking effects in baseline Atlas

We plan to enable handling of additional factors or blocking effects for baseline expression in the near future.

### Atlas data in R

We plan to make baseline expression data available for download as R objects. We will also create an R package in Bioconductor (24) for accessing all Atlas data.

We are always listening to the feedback from our users, and the future plans will be adjusted according to the user requirements.

## SUPPLEMENTARY DATA

Supplementary Data are available at NAR Online.
